# Olive Leaf, Thyme and Green Walnut Husk Extracts as Functional Ingredients in Dairy Products: A Review

**DOI:** 10.17113/ftb.64.03.26.9519

**Published:** 2026-09-15

**Authors:** Elizabeta Zandona, Vedrana Pleš, Silvija Pavković, Ines Cindrić, Almir Abdurramani, Vesna Zechner-Krpan, Irena Barukčić Jurina, Marijana Blažić

**Affiliations:** 1 Karlovac University of Applied Sciences, Trg J. J. Strossmayera 9, 47000 Karlovac, Croatia; 2 Faculty of Food Technology, Josip Juraj Strossmayer University of Osijek, Franje Kuhača 18, 31000 Osijek, Croatia; 3 Faculty of Food Technology and Biotechnology, University of Zagreb, Pierottijeva 6, 10000 Zagreb, Croatia

**Keywords:** olive leaf, thyme, green walnut husk, polyphenols, dairy products, functional ingredients

## Abstract

Oxidative stress and the associated inflammation play key roles in the development of many chronic diseases. As alternatives to synthetic antioxidants, plant-derived materials with potential anti-inflammatory and antioxidant properties, such as olive (*Olea europaea*) leaves, thyme (*Thymus vulgaris*) and green walnut (*Juglans regia*) husks, have attracted increasing scientific interest. These materials are rich in phenolic compounds, including oleuropein and hydroxytyrosol in olive leaves, thymol and rosmarinic acid in thyme, and ellagitannins and juglone in green walnut husks. Numerous studies have demonstrated the strong antioxidant activity of these extracts using analytical assays such as DPPH, FRAP and ABTS. However, such *in vitro* antioxidant capacity does not necessarily translate directly into biological efficacy *in vivo*, as polyphenol stability, release, metabolism and bioavailability may be strongly influenced by interactions with proteins, lipids and other components of dairy matrices. Their incorporation into dairy products has shown promising technological effects, including improved oxidative stability, reduced lipid oxidation, delayed microbial spoilage and potential shelf-life extension. Reported application contents vary substantially among studies and product types, including 0.4–5 % for olive leaf extract in yogurt systems, 0.4–0.8 % for thyme extract in yogurt, 0.1–1 mg/mL for thyme extract in milk systems, 0.5–3 % for olive leaf extract in cheeses, and 100–200 mg/kg for green walnut husk extract in cream-based products. However, these values should be interpreted cautiously because extract composition, matrix type and units of expression differ among studies. Nevertheless, evidence supporting direct health benefits in humans remains limited, and further *in vivo* and human intervention studies are needed to confirm their physiological relevance. This review summarises the polyphenolic profiles of the olive leaf, thyme and green walnut husk extracts, their technological and sensory effects in dairy matrices, and advances in sustainable extraction methods. Overall, the available findings support their potential as natural, clean-label ingredients for innovative dairy products, primarily from a technological and functional formulation perspective.

## INTRODUCTION

The growing interest in novel functional foods and naturally derived bioactive ingredients has led to intensified research on plant-based compounds with potential health-promoting effects. Oxidative stress, defined as an imbalance between antioxidant capacity and reactive oxygen species formation, plays a significant role in the development of chronic diseases. Since synthetic antioxidants have limitations and may cause side effects, consumers are increasingly demanding clean label or natural foods that are minimally processed. In addition, the need to reduce the environmental impact of the food industry through sustainable production and circular economy strategies has increased interest in the valorisation of agro-industrial by-products, such as olive leaves and green walnut husks, as sources of high-value bioactive compounds (*1*-*3*).

Among such natural sources, extracts of olive leaf, thyme, and green walnut husk are particularly rich in polyphenolic components. These raw materials are especially relevant from a sustainability perspective, as they largely originate from by-products of the food industry and therefore represent an underutilised resource with high potential for value-added applications. In addition to being investigated in both *in vitro* and *in vivo* systems, their incorporation into food products, especially those with added functional value, is increasingly being explored. Dairy products such as yogurt, cheese and fermented beverages are considered suitable carriers for such bioactive ingredients due to their widespread consumption, favourable technological compatibility, and the fact that they are naturally low in polyphenolic compounds, which makes their enrichment particularly meaningful. Furthermore, functional dairy products belong to the most frequently consumed category of functional foods, highlighting their relevance for the delivery of plant-derived antioxidants and other beneficial constituents. Incorporating such extracts into dairy products may enhance their stability, improve nutritional and functional properties, and potentially contribute to health-promoting effects (*4*). Although several reviews have addressed plant-derived polyphenols, natural antioxidants or functional dairy products in general, there is still a lack of focused synthesis comparing olive leaf, thyme and green walnut husk extracts specifically in the context of dairy matrices. In particular, limited attention has been given to the relationship between their phytochemical composition, extraction approaches, technological performance, sensory limitations, regulatory aspects and the level of evidence supporting their functionality in real dairy products. Therefore, this review provides an overview of the polyphenolic profiles and antioxidant properties of olive leaf, thyme and green walnut husk extracts, as well as their technological performance, sensory implications and potential applications in functional dairy products.

## REVIEW METHODOLOGY

This narrative review was prepared based on a literature search conducted in scientific databases including Web of Science, Scopus, PubMed, ScienceDirect and Google Scholar. The search focused primarily on publications from 2000 to 2026, with particular emphasis on recent studies published during the last ten years. The following keywords and their combinations were used: “olive leaf extract”, “thyme extract”, “green walnut husk extract”, “polyphenols”, “phenolic compounds”, “antioxidant activity”, “antimicrobial activity”, “dairy products”, “yogurt/yoghurt”, “cheese”, “fermented milk”, “milk matrix”, “functional foods”, “green extraction” and “sustainable extraction”. Relevant original research articles, reviews, book chapters and regulatory documents were considered. Priority was given to studies directly addressing the phytochemical composition, extraction methods, biological activity and application of olive leaf, thyme and green walnut husk extracts in dairy or food matrices. Studies not directly related to plant polyphenols, dairy applications or the selected plant materials were excluded from the main discussion.

## PHYTOCHEMICAL COMPOSITION AND BIOLOGICAL ACTIVITY

### Olive leaf (*Olea europaea* L.)

Olive (*Olea europaea* L.) leaves are generated as a by-product during olive harvesting and olive oil production. It is estimated that olive leaves account for more than 10 % of the total harvested olive mass (*5*). As an evergreen species, the olive tree provides a continuous supply of leaves throughout the year. This represents a significant advantage, as the availability of many plant materials is often limited by seasonal factors. Consequently, olive leaves represent a sustainable and widely available source of bioactive compounds.

Numerous studies have shown that olive leaf extracts are rich in phytochemicals with various biological activities. The bioactive compounds present in olive leaves can be classified into five major groups: secoiridoids (oleuropein, demethyloleuropein, verbascoside and ligstroside), flavones (luteolin, diosmetin, luteolin-7-glucoside, apigenin-7-glucoside and diosmetin-7-glucoside), flavonols (rutin, quercetin and kaempferol), flavan-3-ols (catechin) and substituted phenols (tyrosol, hydroxytyrosol, vanillin, vanillic acid and caffeic acid) (Fig. 1 (*4*)) (*6*). Hydroxytyrosol, one of the most potent natural antioxidants, is present in olive leaves both in free form and as a hydrolysis product of oleuropein. During enzymatic or chemical hydrolysis, the ester bond between the elenolic acid moiety and hydroxytyrosol in the oleuropein molecule is cleaved, releasing hydroxytyrosol into the extract (*7*). This process can occur naturally during leaf senescence, fermentation, or because of processing conditions such as pH changes, heat treatment or enzymatic action (*e.g*. β-glucosidases and esterases). Consequently, olive leaf extracts obtained through extraction methods that promote hydrolysis (*e.g*. acidic hydrolysis, prolonged heating or fermentation) may contain significantly higher concentrations of hydroxytyrosol than fresh leaf extracts. Hydroxytyrosol exhibits strong radical scavenging activity, metal-chelating ability and antimicrobial effects, contributing synergistically to the overall antioxidant and health-promoting properties of olive leaf extracts (*5*).

**Fig. 1:**
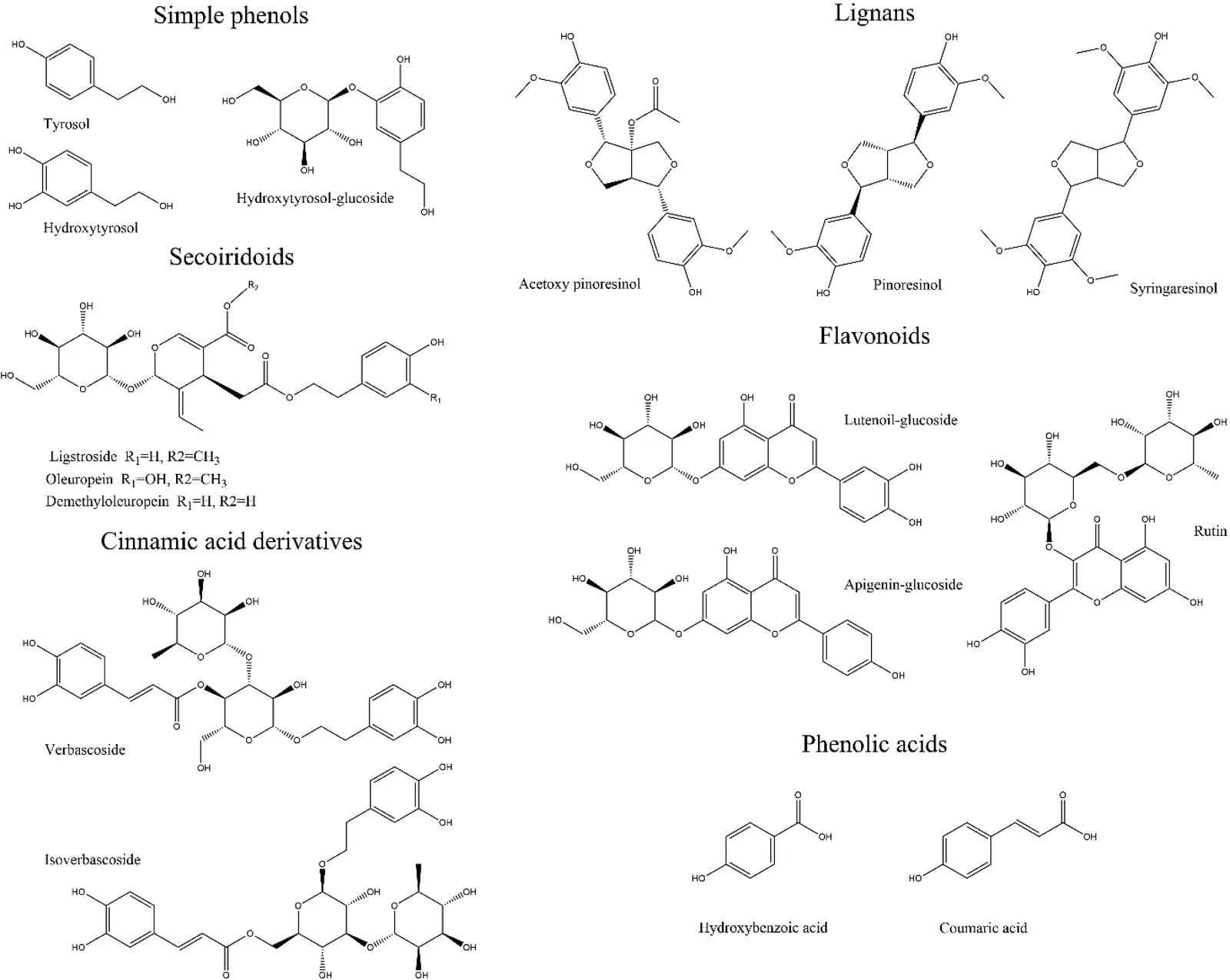
Phenolic classes and structures of main phenolic compounds in olive leaves (*4*)

Its biological activity, together with that of oleuropein, has been associated with various health benefits, including anti-inflammatory, cardioprotective and neuroprotective effects, largely attributed to their combined ability to scavenge free radicals, modulate enzyme activity and inhibit the proliferation of pathogenic microorganisms (*5*).

### Thyme (*Thymus vulgaris* L.)

Thyme (*Thymus vulgaris* L.) is a Mediterranean perennial aromatic plant from the Lamiaceae family, widely used as a culinary and medicinal herb due to its characteristic aroma and high content of bioactive compounds. From a sustainability perspective, thyme is relevant because it is widely cultivated, easily dried and processed, and available as a stable plant material suitable for extraction and incorporation into food formulations. In addition to its essential oil constituents, thyme is an important source of phenolic compounds, particularly phenolic acids and flavonoids, which contribute to its antioxidant, antimicrobial and anti-inflammatory potential.

The main phenolic acids reported in thyme extracts include rosmarinic, caffeic, ferulic and *p*-coumaric acids, while the flavonoid fraction includes compounds such as luteolin, apigenin, quercetin and kaempferol (Fig. 2) (*8*). Among these, rosmarinic acid is generally considered one of the most abundant and functionally relevant phenolic compounds in thyme extracts. It contributes to antioxidant activity through radical scavenging and metal-chelating mechanisms and may also modulate inflammatory and microbial processes (*8*). Thyme also contains phenolic monoterpenes such as thymol and carvacrol, which are mainly associated with the essential oil fraction and are particularly relevant for antimicrobial activity. Therefore, when discussing thyme extracts in the context of functional dairy products, it is important to distinguish between the polyphenolic fraction, which is mainly responsible for antioxidant capacity, and the essential oil-related constituents, which contribute strongly to antimicrobial and sensory properties.

**Fig. 2:**
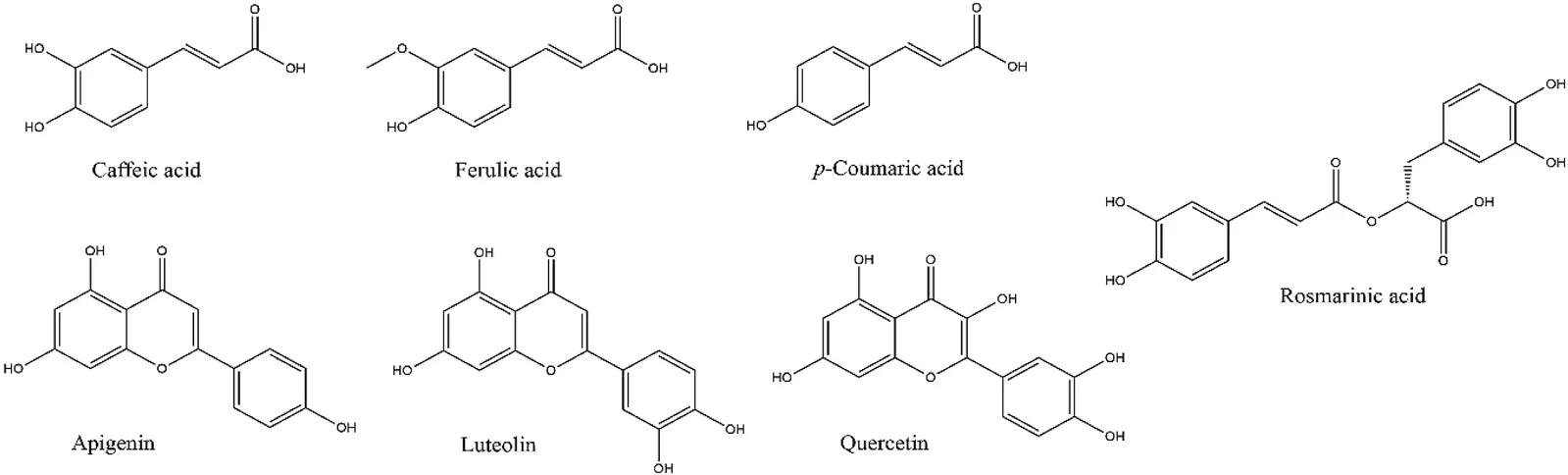
Structural representation of selected phenolic compounds commonly reported in thyme extracts: rosmarinic acid, caffeic acid, ferulic acid, *p*-coumaric acid, luteolin, quercetin and apigenin (*8*)

### Green walnut (*Juglans regia* L.) husk

The green walnut (*Juglans regia* L.) husk is the outer pericarp layer of the walnut fruit, generated as a by-product during walnut harvesting and processing. It represents a significant portion of agricultural waste, often discarded despite its rich phytochemical composition. Recent studies have highlighted the potential of green walnut husks as a valuable source of bioactive compounds, particularly polyphenols, which exhibit strong antioxidant, antimicrobial and anti-inflammatory properties (*9*, *10*). As walnut trees are widely cultivated and their fruits harvested annually, the green husk is seasonally abundant and offers opportunities for the sustainable valorisation of agro-industrial by-products (*10*). The phytochemical profile of green walnut husks includes various classes of phenolic compounds, such as phenolic acids (gallic, *p*-coumaric and ellagic acids), naphthoquinones (juglone), flavonoids (quercetin, myricetin and catechin) and tannins (*9*, *10*) (Fig. 3). Although the main focus of this review is on phenolic compounds, Fig. 3 also includes selected non-phenolic bioactive constituents reported in walnut-derived materials, such as tocopherols and fatty acids, because they may contribute to the overall bioactive profile and oxidative stability of walnut-based extracts. Among these, juglone is a characteristic compound of walnut species and is known for its antimicrobial and allelopathic activity. Gallic and ellagic acids are the most prevalent phenolic acids, contributing significantly to the antioxidant potential of the husk. These compounds act through multiple mechanisms, including free radical scavenging, metal ion chelation, and inhibition of oxidative enzymes, which makes green walnut husk a promising candidate for use in food preservation, cosmetics, and nutraceutical applications (*11*).

**Fig. 3:**
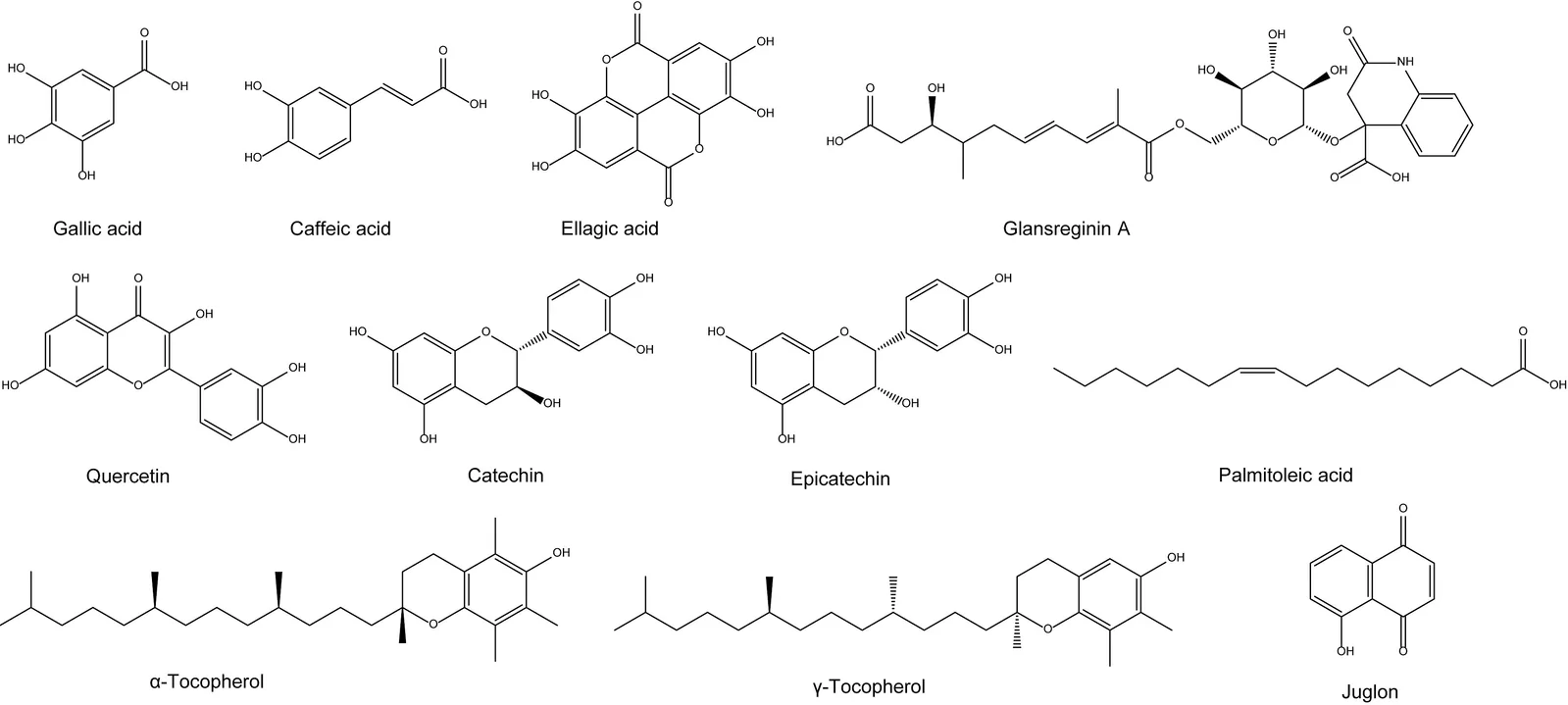
Structural representation of selected phytochemicals reported in walnut-derived materials, including phenolic and non-phenolic bioactive compounds, adapted from (*10*)

## SUSTAINABLE TECHNIQUES FOR EXTRACTING BIOACTIVE COMPOUNDS

Efficient extraction of bioactive compounds such as polyphenols is a critical step in the valorisation of plant-based materials. Conventional extraction methods are widely used due to their simplicity and reproducibility. These include solid–liquid extraction, Soxhlet extraction, maceration and percolation, most often performed with organic solvents such as ethanol, methanol or acetone. Maceration, based on prolonged soaking of plant material at ambient temperature, offers a low-cost and accessible option but often requires long extraction times and may result in lower yields. Percolation, on the other hand, allows continuous solvent flow through the sample, improving mass transfer and recovery efficiency. Soxhlet extraction, although widely recognised for its effectiveness, involves elevated temperatures and large solvent volumes, which raises safety and environmental concerns. Additionally, these methods are generally time-consuming, energy-intensive and associated with high solvent consumption, which can compromise both sustainability and the stability of heat-sensitive compounds (*12*-*14*). To address the limitations of conventional techniques, increasing attention has been directed towards green and innovative extraction methods. These modern approaches aim to reduce environmental impact and improve efficiency by shortening extraction time, minimising solvent usage, and preserving the functional integrity of sensitive bioactive molecules. Among the most studied are ultrasound-assisted extraction (UAE), microwave-assisted extraction (MAE), pressurised liquid extraction (PLE) and supercritical fluid extraction (SFE). These techniques offer advantages such as lower energy input, higher selectivity and improved extraction kinetics, particularly when optimised for solvent polarity, temperature and pressure (*15*). Importantly, the choice of extraction technique significantly influences the chemical profile of the extract, including the relative proportions of phenolic acids, flavonoids and other bioactive constituents. This compositional variability affects not only antioxidant and antimicrobial activity but also the functional behaviour of the extract in complex food matrices, determining solubility, stability and potential interactions with proteins and lipids (*10*).

Furthermore, the extraction method can be strategically selected to tailor the extract composition to its intended food application. For instance, when the extract is intended for incorporation into fermented products such as yogurt or kefir, techniques using aqueous or hydroalcoholic solvents, such as UAE or PLE, are preferred, as they provide extracts enriched in water-soluble polyphenols (*e.g*. hydroxytyrosol from olive leaves). These compounds ensure homogeneous distribution and functional stability during fermentation.

Conversely, in the case of cheeses, extraction techniques and solvent systems that yield less polar phenolic fractions may be considered as a potential formulation approach for lipid-rich dairy matrices. However, direct empirical evidence confirming their preferential incorporation and functional performance in the cheese fat matrix remains limited. Such extracts, for example those containing flavonoid aglycones or juglone derivatives from green walnut husks, integrate more effectively into the milk fat matrix and contribute to lipid protection and extended shelf life. Moreover, in the formulation of functional beverages, extracts with high water solubility are preferred, which can be achieved through UAE or maceration in aqueous solvents, while in dietary supplements (capsules or powders), techniques such as MAE or PLE are more suitable due to their ability to yield concentrated and stable extracts with longer shelf life and easier incorporation. Thus, the choice of extraction method determines not only the quantitative yield but also the functional suitability of bioactive compounds for specific food applications.

Although modern extraction methods offer numerous advantages in terms of efficiency, selectivity and sustainability, the choice of an appropriate technique largely depends on the physicochemical properties of the plant matrix and the targeted bioactive compounds. Parameters such as particle size, moisture content, structural complexity, and the polarity and thermal sensitivity of the compounds significantly influence the effectiveness of a given extraction method (*16*). When extracting polyphenolic compounds from olive leaves, UAE has been identified as one of the most efficient techniques. This method is characterised by relatively short extraction times, moderate temperatures that preserve thermolabile components and environmental compatibility, making it one of the most frequently employed techniques for polyphenol recovery from olive leaves. Giacometti et al. (*17*) demonstrated that the total polyphenol and flavonoid contents extracted from olive leaves using UAE were 14.31 and 19.50 % higher, respectively, than those obtained with conventional extraction techniques. In addition to UAE, other frequently used methods with higher efficiency than conventional techniques include MAE and SFE (*18*).

As with other aromatic herbs, the efficiency of extracting bioactive compounds from thyme largely depends on the morphological characteristics of the plant. Leaves and stems differ significantly in their phenolic content, with the leaves containing much higher concentrations of polyphenols and essential oils, particularly monoterpenoid phenols such as thymol and carvacrol, while the stems are poorer in these compounds and thus less suitable for the extraction of high-value components (*19*).

The choice of extraction method has a significant impact on the yield and antioxidant capacity of the extract. According to a study by Palmieri et al. (*20*), Soxhlet extraction was the most effective method for recovering polyphenols from thyme, achieving a yield of (9.25±0.02) % after two hours of extraction. This indicates that 9.25 g polyphenols were extracted from 100 g dry plant material. However, the main drawbacks of this technique are the long extraction time and high temperature, which may contribute to partial degradation of heat-sensitive compounds. As a faster and more energy-efficient alternative, UAE is frequently used. It operates at lower temperatures, requires less solvent and shortens processing time. Although UAE helps preserve thermolabile compounds, the same study reported a significantly lower polyphenol yield of (1.62±0.02) %, more than five times less than Soxhlet extraction. Despite this, UAE is still considered a suitable “green” technology due to its reduced energy use, lower environmental impact and potential for industrial application.

The efficiency of bioactive compound extraction from green walnut husk greatly depends on the selected method, type of solvent and processing conditions. As with other plant matrices, the morphological features and chemical composition of the raw material are crucial for determining the most suitable extraction technique. Given the high content of phenolics and thermolabile antioxidants, there is a growing shift towards “green” extraction technologies that preserve bioactivity while minimising energy and solvent consumption. For the efficient recovery of polyphenols from green walnut husk, UAE and MAE have proven particularly effective. According to Momen and Satari (*21*), combining microwave pre-treatment (MWP) with UAE (MWP–UAE) significantly enhances total antioxidant content, expressed as gallic acid equivalents (GAE) on dry mass basis, achieving 3.64 g/100 g. This represents a notable increase compared to standard UAE (3.32 g/100 g) and maceration (1.96 g/100 g, 24 h). Extraction performance is significantly influenced by factors such as solvent choice (typically ethanol, water, or their mixtures), pre-treatment techniques (*e.g*. ultrasound or microwave) and operational parameters including temperature, time, solid-to-liquid ratio, and energy input. Proper optimisation of these conditions is essential for maximising yield and preserving the functional integrity of the extracts. As previously demonstrated, the application of innovative and green extraction techniques significantly reduces the environmental footprint of the process, thereby contributing to the sustainable valorisation of agro-industrial waste such as olive leaves and green walnut husks. Beyond improving extraction efficiency, these technologies also foster circular economy principles, reduce waste generation and create high-value ingredients for the food and pharmaceutical industries (*22*). Therefore, green extraction methods not only enhance selectivity and efficiency but also represent an environmentally friendly and socially responsible approach to the industrial processing of by-products.

## ANTIOXIDANT ACTIVITY OF POLYPHENOLS FROM OLIVE LEAF, THYME AND GREEN WALNUT HUSK EXTRACTS

Phenolic compounds from olive leaf, thyme and green walnut husk extracts, particularly oleuropein, rosmarinic acid and juglone, exhibit pronounced antioxidant potential that, in dairy systems as well as in food matrices in general, results in the inhibition of oxidative reactions and limitation of microbial growth, thereby directly contributing to shelf-life extension. Their chemical structures, characterised by *ortho*-diphenolic and quinone groups, enable them to act through transition metal chelation, neutralisation of reactive oxygen species, and inhibition of oxidative enzymes. This reduces hydroxyl radical formation and interrupts lipid peroxidation chain reactions, resulting in the preservation of lipids, proteins and other sensitive food constituents.

The antimicrobial effects of these compounds are closely related to their antioxidant activity. By neutralising reactive species and altering redox balance, polyphenols interfere with microbial metabolic processes, while metal chelation reduces the availability of essential cations required for microbial proliferation. Juglone, due to its quinone structure, can induce redox perturbations in microbial cells and compromise membrane integrity and protein function, as demonstrated for *Listeria monocytogenes* ATCC 19115, thereby contributing to the antimicrobial potential of green walnut husk extracts (*23*). Oleuropein and rosmarinic acid inhibit enzymes such as lipoxygenase, which participate in oxidative reactions within food matrices, thereby preventing the formation of undesirable oxidation products responsible for off-flavours and deterioration of sensory quality (*24*, *25*). The structural formulae of oleuropein, rosmarinic acid and juglone, which illustrate their key functional groups responsible for these mechanisms, are shown in Fig. 1, Fig. 2 and Fig. 3.

The combined effects of radical scavenging, metal chelation, and enzyme inhibition provide multilayered protection against both oxidative and microbiological degradation. As a result, polyphenolic extracts not only minimise the risk of oxidative rancidity, discolouration and nutrient loss but also function as natural preservatives that slow down the growth of spoilage microorganisms (*23*). These properties highlight oleuropein, rosmarinic acid and juglone as particularly valuable for application in food technology, with strong potential to replace or complement synthetic antioxidants and preservatives, thereby improving product stability, safety and shelf-life. Radical scavenging mechanisms of polyphenols from olive leaf, thyme and walnut husk share core antioxidant actions, but have distinctive features based on their molecular structures (Table 1 (*26*-*28*)). In essence, all polyphenols scavenge radicals chiefly by donating hydrogen atoms or electrons, but olive leaf polyphenols have outstanding radical-trapping efficiency due to their chemical structure, thyme compounds add metal chelation effects that enhance antioxidant protection, and walnut husk polyphenols combine radical scavenging with redox cycling typically associated with quinones (*26*-*28*). In milk matrices these antioxidant mechanisms can be further modulated by interactions with milk proteins and lipids, which may alter extract efficacy and biostability. Polyphenols bind non-covalently to milk proteins *via* hydrogen bonds, hydrophobic interactions and sometimes covalent bonds, stabilising polyphenols and protecting them from degradation during storage and digestion (*29*, *30*). These interactions can affect the release and absorption of polyphenols in the gastrointestinal tract, sometimes reducing bioavailability but often improving stability through digestion (*29*, *31*). In addition, the complexes may influence the texture and sensory properties of food products and affect their functional and health-promoting qualities (*30*, *32*).

**Table 1:** Comparative analysis of antioxidant mechanisms and analytical assessment of selected polyphenols from olive leaf extract, thyme extract and green walnut husk extract (*26*-*28*)

Feature	Olive leaf	Thyme	Walnut husk
Main representative compounds	Oleuropein, hydroxytyrosol, verbascoside, luteolin derivatives	Rosmarinic acid, caffeic acid, ferulic acid, luteolin, apigenin	Ellagic acid, gallic acid, catechin, quercetin, myricetin, juglone
Common analytical assays used to evaluate antioxidant activity	DPPH, ABTS, FRAP, ORAC and related *in vitro* antioxidant capacity assays	DPPH, ABTS, FRAP and related radical-scavenging assays	DPPH, ABTS, FRAP and total antioxidant capacity assays
Main antioxidant mechanisms	H-atom donation, phenoxyl radical stabilisation	Electron transfer, H-atom donation, metal chelation	H-atom transfer, redox cycling
Biologically relevant oxidative targets/processes	ROS neutralisation, inhibition of lipid peroxidation, protection of phenolic compounds from oxidative degradation	ROS neutralisation, inhibition of lipid oxidation, reduction of oxidative enzyme activity	ROS modulation, inhibition of lipid oxidation, redox-related antimicrobial contribution
Key functional groups	Catechol structure in oleuropein	Hydroxyl groups on flavonoid rings	Multiple hydroxyl groups, quinone
Lipid peroxidation inhibition	Strong, especially in membranes	Moderate to strong	Strong inhibition
Metal chelation	Moderate	Significant	Less emphasised
Overall radical-scavenging capacity	High stoichiometric capacity	Correlated with phenolic content	High due to multiple -OH groups

DPPH=2,2-diphenyl-1-picrylhydrazyl, ABTS=2,2′-azino-bis(3-ethylbenzothiazoline-6-sulfonic acid), FRAP=ferric reducing antioxidant power, ORAC=oxygen radical absorbance capacity, ROS=reactive oxygen species. DPPH and ABTS are synthetic analytical probes used to estimate antioxidant capacity *in vitro* and should not be interpreted as biologically occurring radicals

## TECHNOLOGICAL AND FUNCTIONAL EFFECTS OF POLYPHENOLS IN DAIRY PRODUCTS

### Role of dairy matrices in the delivery of plant-derived polyphenols

Dairy products serve as effective carriers for plant-derived polyphenols due to their balanced composition of proteins, fats and carbohydrates, which can stabilise bioactive compounds and influence their release during digestion. Fermented and non-fermented dairy matrices such as yogurt, kefir, cheese and milk-based beverages offer favourable physicochemical environments for the incorporation of polyphenols and antioxidants, supporting their protection from oxidation, improved solubility and potentially enhanced bioavailability (*33*). Integrating plant extracts into dairy systems may enhance nutritional value, slow oxidative processes and improve microbiological stability. However, such fortification must be carefully optimised to avoid adverse effects on sensory and textural properties (*33*, *34*). Olive leaf extract, thyme extract and green walnut husk extract (GWHE) demonstrate strong potential as multifunctional ingredients in dairy products. When applied at optimised doses, they can significantly improve oxidative stability, microbial safety and nutritional properties while maintaining acceptable sensory properties. These extracts therefore represent promising natural tools for developing innovative, health-promoting and sustainable dairy formulations (*35*).

The interactions between polyphenols and dairy components are complex and highly dependent on the molecular characteristics of both the polyphenols and the matrix. Understanding these mechanisms is essential for optimising extraction, formulation and processing strategies aimed at developing stable, palatable, and nutritionally enriched dairy products with improved bioactive functionality.

### Binding and interaction mechanisms between polyphenols and milk components

Polyphenols are highly reactive bioactive compounds capable of interacting with various components of the dairy matrix, including proteins, lipids and carbohydrates. These interactions occur through non-covalent forces such as hydrogen bonding, hydrophobic interactions and van der Waals forces, as well as through covalent bonding under certain conditions. The chemical nature and strength of these interactions determine not only the stability and bioavailability of polyphenols but also the physicochemical and sensory properties of the final dairy product (*29*).

Among milk proteins, caseins and whey proteins (mainly β-lactoglobulin and α-lactalbumin) exhibit a strong affinity for polyphenols (*36*). Due to their flexible structure and high proline content, caseins readily form complexes with flavonoids such as catechin, quercetin and epigallocatechin gallate. Covalent interactions can also occur between hydroxyl groups of polyphenols and thiol or amino groups of proteins, resulting in stable protein–polyphenol conjugates (*36*). For example, quercetin can form covalent bonds with thiol groups of cysteine residues, leading to modified protein functionality and improved antioxidant stability. Casein–polyphenol complexes have been shown to influence the textural and optical properties of fermented dairy products, contributing to increased viscosity and turbidity, and, in some cases, to a more compact gel network.

The formation of such complexes may have both beneficial and adverse effects. On the one hand, polyphenol–protein binding can enhance the stability of polyphenols during digestion by protecting them from oxidation or enzymatic degradation, potentially prolonging their antioxidant activity in the gastrointestinal tract (*37*). On the other hand, excessive binding may reduce the proportion of free, absorbable polyphenols, thus lowering their bioavailability. These dual effects are influenced by factors such as pH, ionic strength, temperature, and the polyphenol-to-protein ratio (*30*, *38*, *39*). From a technological standpoint, processing conditions, particularly heat treatment, play a critical role in modulating interactions between polyphenols and milk components. Thermal denaturation of whey proteins exposes hydrophobic regions and reactive thiol groups, increasing their capacity to bind polyphenols. While this can improve encapsulation efficiency and antioxidant retention, high temperatures may also lead to degradation or polymerisation of heat-sensitive polyphenols (*35*). Optimising temperature and processing time is therefore essential to balance protein unfolding and polyphenol preservation (*40*). In dairy fermentation, these interactions may influence acidification kinetics, gel firmness and microstructure, all of which are critical for product texture and stability. Recent studies have also explored the potential of casein micelles and whey protein isolates as natural carriers for the microencapsulation of polyphenols. These protein-based delivery systems can improve solubility, control release, and protect phenolic compounds from degradation during storage and digestion. Casein micelles exhibit strong potential for encapsulating lipophilic polyphenols due to their amphiphilic nature and dynamic structural properties. The formation of casein–polyphenol nanoparticles can therefore enhance the functional and nutritional value of fortified dairy products while maintaining desirable texture and sensory quality (*30*).

### Impact on fermentation processes

Polyphenols can influence fermentation kinetics by interacting with starter cultures and milk components. Olive leaf extract maintains the viability of *Lactobacillus* and *Streptococcus* spp., but higher contents can slow acidification or increase syneresis (*41*, *42*). Phenolic monoterpenes in thyme extract may inhibit spoilage organisms, yet at high concentrations they can suppress starter culture growth (*43*), while the potent antimicrobial activity of green walnut husk extract may require content adjustment to avoid interference with fermentation. Careful evaluation of extract concentration is essential to preserve fermentation performance and product consistency.

### Effects on texture, rheology and microstructure

Polyphenols can bind to milk proteins, altering gel structure and rheology. Casein–polyphenol complexes may increase gel firmness, enhance viscosity in stirred yogurts, influence water-holding capacity and syneresis, and strengthen the microstructure in cheeses. Syneresis is an important quality parameter in fermented dairy products because it reflects the ability of the gel network to retain serum during storage. Polyphenol–protein interactions may reduce syneresis by strengthening the casein network and improving water-holding capacity. However, at higher extract concentrations, excessive protein aggregation or disruption of the gel structure may have the opposite effect and increase serum separation. The specific effects depend on the extract type, its concentration, the molecular mass of polyphenols, and the fat content of the product. Rheological studies have shown that the incorporation of polyphenol-rich extracts into yogurt or cheese can increase both the storage (G′) and loss (G″) moduli, indicating the formation of stronger and more elastic gel networks. Nevertheless, aggregation or phase separation may occur at higher polyphenol concentrations. According to Lorenzen et al. (*44*), milk fat content significantly influences the microstructure and rheological properties of rennet casein gel emulsions, thereby affecting gel network formation and texture. Conversely, low-fat and fat-free products tend to exhibit reduced viscosity and weaker gel structures (*45*), which often necessitates the addition of milk solids or fat replacers such as inulin, maltodextrin, starch, or whey protein concentrates to achieve desirable textural and sensory attributes (*40*). Furthermore, the fortification of dairy products with fruits and herbal extracts rich in polyphenols has been shown to modify rheological and sensory properties. Dabija et al. (*46*) observed that yogurts enriched with plant extracts exhibited increased viscosity, improved consistency and thixotropic properties, attributed to protein–phenolic interactions within the gel matrix. However, such formulations also present challenges, including the maintenance of the quality of natural ingredients, ensuring microbial stability and achieving consumer acceptance.

### Sensory impact and formulation challenges

Olive leaf, thyme and green walnut husk extracts are of particular interest because of their high polyphenolic content, diversity of bioactive constituents and natural origin, making them ideal candidates for clean-label functional dairy innovations. Although polyphenols may contribute to desirable flavour notes at low concentrations, higher concentrations often result in bitterness and astringency (especially olive leaf and walnut husk extracts), darkening or green–brown discolouration, herbal or medicinal off-flavours, and changes in texture or mouthfeel. Perception thresholds for bitterness and astringency depend strongly on the specific phenolic compound, its concentration, the dairy matrix and interactions with proteins and fat. Although compounds such as oleuropein, tannins and some phenolic acids are associated with bitter or astringent sensations, standardised threshold values for olive leaf, thyme and green walnut husk extracts in dairy products remain limited. Sensory optimisation should therefore be performed for each extract–product combination rather than relying only on total phenolic content. For example, the addition of olive leaf extract to cheese at higher concentrations results in pronounced bitter notes that reduce sensory acceptability, while lower concentrations have minimal impact on the taste and colour of the product (*34*).

The sensory properties of fortified dairy products are critical for consumer acceptance, so an optimal extract dose is crucial to preserving sensory characteristics without compromising functional properties. Polyphenol interactions with milk proteins can mitigate these undesirable attributes, improving overall palatability. The presence of milk fat further contributes to flavour masking by incorporating hydrophobic polyphenols into the lipid phase, thereby reducing their perception on the palate. Additionally, polyphenol–lipid interactions can enhance oxidative stability, as polyphenols tend to localise at the lipid–water interface and inhibit lipid peroxidation, thus extending the shelf life of the product.

## APPLICATION OF OLIVE LEAF, THYME AND GREEN WALNUT HUSK EXTRACTS IN SPECIFIC DAIRY PRODUCTS

### Yogurt and fermented milk beverages

Fermented dairy products are widely recognised as effective carriers of bioactive compounds due to their high nutritional value, probiotic potential and physiological benefits. Incorporating polyphenol-rich extracts into yogurt and fermented milk offers opportunities to enhance antioxidant capacity, improve microbial stability and create value‐added functional foods. These systems also provide a suitable environment for examining how plant-derived polyphenols interact with starter cultures, milk proteins and fermentation dynamics, thereby influencing both technological performance and sensory quality. Table 2 (*35*, *41*-*43*, *47*-*53*) summarises the effects of olive leaf, thyme and green walnut husk extracts in yogurt, fermented milk and pasteurised milk systems.

**Table 2:** Application of olive leaf, thyme and green walnut husk extracts in yogurts, fermented milk and pasteurised milk

Extract type	Matrix	Extract dose	Main observed effect	Reference
Olive leaf (aqueous)	Yogurt	1.5, 3 and 5 % (*V*/*V*)	Maintained probiotic viability, increased antioxidant activity, no negative effect on sensory acceptance	(*41*)
Olive leaf (aqueous)	Yogurt (cow, sheep, goat)	0.5, 1.0, 1.5 and 2.0 % (*m*/*V*)	Lower pH, improved antioxidant capacity, increased cohesiveness	(*43*)
Olive leaf (aqueous)	Yogurt	Spraying 0.4 and 0.6 % (*m*/*V*) solution after fermentation	Reduced bitterness, antimicrobial effect (control of spoilage microbiota)	(*42*)
Olive leaf (aqueous)	Yogurt	Adding 0.4 and 0.6 % (*m*/*V*) to milk before fermentation	Higher total phenolic content, enhanced antioxidant properties	(*47*)
Thyme (ethanolic)	Set yogurt	0.4–0.8 % (*m*/*V*)	Improved flavour and overall acceptability, reduced pH and titratable acidity; suppressed yeast and mould growth	(*43*)
Thyme (aqueous)	Pasteurised milk	0.5 mg/mL	Increased total phenolic content and antioxidant activity	(*49*)
Thyme (aqueous)	Raw milk and pasteurised milk	0.1, 0.5 and 1 mg/mL	Improved microbial quality of pasteurised milk (reduced total bacterial count) and increased antioxidant activity due to higher phenolic content, good sensory acceptability	(*50*)
Green walnut fruit and leaf extract	*Kurut* (fermented, then salted and dried)	5, 10 and 15 g	Inhibition of *Enterobacteriaceae* and *Staphylococcus*–*Micrococcus* groups at higher extract doses, linked to phenolic content and flavonoids	(*48*)
Walnut	Yogurt	0, 1, 2.5, 3.5 and 5 %	Increased phenolic content and antioxidant activity, higher pH and lower titratable acidity during storage, reduced syneresis, highest sensory acceptability at 1 % addition	(*51*)
Walnut kernel extract	Yogurt	0, 10, 20 and 30 %	Reduced pH and increased titratable acidity during storage; slight decreases in fat and total solids, consistently lower syneresis; significant increase in total phenolics, decline in DPPH activity over storage	(*52*)
Green walnut husk (ethanolic)	Model systems	≥0.25×MIC	High antioxidant activity, increased phenolic content, reduced total plate count and yeast/mould counts	(*35*)
Green walnut husk (methanolic)	Table cream (22–26 % milk fat)	0, 100 and 200 mg/kg	Lower peroxide and acidity values; inhibited total, lipolytic and proteolytic bacteria growth; enhanced sensory acceptance	(*53*)

Extract dose units are presented as reported in the original studies. Due to the heterogeneity of units, extract composition, product matrices and modes of application, the reported doses should not be directly compared among studies. MIC=minimum inhibitory concentration

The studies consistently show that moderate volume fractions of olive leaf extract improve antioxidant capacity and microbiological stability while maintaining acceptable sensory properties, particularly when applied at φ=1–3 % (*41*, *42*, *47*). Application timing plays a notable role: surface treatment after fermentation minimises bitterness, whereas pre-fermentation addition enhances phenolic content but increases the risk of sensory defects.

Thyme extract demonstrates strong antimicrobial effects against yeasts and moulds and contributes to desirable flavour enhancement at low inclusion concentrations. However, higher concentrations may negatively affect starter culture activity, highlighting the importance of dose optimisation.

In contrast, direct incorporation of GWHE into conventional yogurt or fermented milk has not yet been reported in the literature. Existing work is limited to model dairy systems, where GWHE demonstrates strong antimicrobial and antioxidant activity, and a single fermented dairy analogue, *kurut*, in which green walnut fruit and leaf extracts reduced *Enterobacteriaceae* and *Staphylococcus*–*Micrococcus* populations (*48*). Although *kurut* differs structurally from stirred or set yogurt, its fermented milk base provides a relevant technological comparison. Additional insights come from studies enriching yogurt with walnut kernels or kernel extracts, which increase total phenolics and antioxidant activity and influence physicochemical parameters such as pH and serum separation. While kernels differ from green husk material, these findings support the technological feasibility of incorporating walnut-derived phenolic fractions into fermented dairy systems. Given the established bioactivity of GWHE in non-dairy foods and model systems, and the demonstrated antioxidant, antimicrobial and technological effects of walnut-derived ingredients in yogurt, GWHE remains a promising but underexplored candidate for functional fermented dairy applications. Its inclusion in Table 2 therefore reflects its technological potential, even though direct yogurt applications are not yet documented.

Collectively, the results indicate that all three extracts offer functional benefits in fermented dairy matrices, but successful application depends on balancing technological advantages with sensory acceptability.

### Cheese

Cheese represents a structurally complex and compositionally diverse dairy matrix in which the incorporation of plant-derived polyphenols can exert pronounced technological and functional effects. Owing to their higher fat content, lower water activity and prolonged ripening periods, cheeses provide an environment in which polyphenols can interact with lipids, proteins and the developing microflora in ways that differ substantially from fermented milk systems. These interactions may enhance oxidative stability, reduce microbial spoilage and improve shelf-life, while also influencing textural characteristics, colour development and flavour complexity. When formulated at appropriate concentrations, extracts such as olive leaf, thyme and green walnut husk can contribute to the creation of clean-label, functional cheese products with improved nutritional and technological attributes.

The studies summarised in Table 3 (*34*, *54*-*59*) highlight clear and consistent effects of plant extracts, primarily olive leaf extract (OLE) and thyme powder, on the nutritional, antioxidant, textural and microbiological properties of various cheese types. Across cheese matrices, OLE emerges as the most extensively investigated ingredient, demonstrating strong potential as a natural functional fortifier. In both Cantal-type and semi-hard cheeses, OLE supplementation consistently increased total phenolic content, flavonoids and antioxidant activity, confirming its high bioactive retention within the cheese matrix. These enhancements were generally dose-dependent, with higher OLE contents yielding more pronounced improvements.

**Table 3:** Application of olive leaf extract and thyme extract in cheese

Extract type	Matrix	Extract dose	Main observed effect	Reference
Olive leaf (aqueous)	Cantal cheese	0.0, 1.0, 2.0 and 3.0 % (*m*/*m*)	Increased phenolic content and antioxidant activity, inhibitory effect on pathogenic bacteria, changes in cheese colour	(*54*)
Olive leaf (aqueous, spray-dried)	Cantal cheese	0.0, 1.0, 2.0 and 3.0 % (*m*/*m*)	Improved antioxidant capacity and microbial stability; increased protein and dry matter; reduced fat, chloride and mesophilic counts; enhanced hardness and cohesiveness while reducing elasticity	(*55*)
Olive leaf (aqueous)	Semi-hard cheese and whey	Determined as GAE: 11.5, 16.6 and 26.3 mg/L	Higher phenolics, antioxidants, fat, protein and solids; firmer texture with reduced elasticity when added before rennet coagulation; good phenol retention; high concentrations caused colour changes; no effect on acidity, water activity or yield	(*56*)
Olive leaf (aqueous)	Semi-hard cheese	0 and 15 % (*V*/*V*) in WPI-based coating (total phenol concentration determined as GAE: 0, 26.3 and 5.94 mg/L	Increased antioxidant potential and phenolic retention; slightly reduced microbial growth; maintained cohesiveness; moderated proteolysis; higher total solids and fat *vs* control; reduced colour deviation during ripening	(*34*)
Olive leaf powder	Fresh cheese	0.5, 1 and 1.5 %	Increased phenolics (54–65 %) and antioxidant activity; minor changes in colour, fat and ash; sensory impact evident, with best acceptance at 0.5 %	(*57*)
Thyme (ethanolic)	White soft cheese	0, 0.5, 1.0 and 1.5 %	Significantly increased antioxidant capacity; microbiological quality improved with reduced aerobic mesophilic and psychrophilic bacteria counts proportional to thyme extract concentration; extended shelf life, and maintained or enhanced sensory quality	(*58*)
Thyme powder	White Syrian cheese	1 and 2 %	Higher thyme powder levels (2 %) increased total solids and salt uptake; samples showed higher acidity during storage; ripening slowed at higher salt concentrations and higher powder levels due to reduced protein degradation and lower soluble nitrogen	(*59*)

Extract dose units are presented as reported in the original studies. Due to the heterogeneity of units, extract composition, product matrices and modes of application, the reported doses should not be directly compared among studies. WPI=whey protein isolate, GAE=gallic acid equivalents

Technologically, the mode and timing of OLE application played a significant role. When incorporated before rennet coagulation, OLE altered the paracasein network, producing firmer curds with increased hardness, gumminess and cohesiveness, accompanied by reduced elasticity (*56*). In spray-dried or whey-based coating formats, OLE also promoted stability during ripening, with coatings improving proteolysis control, fat and total solids retention, and minimising colour deviation. Importantly, none of the OLE treatments negatively affected acidity, water activity or cheese yield, supporting its technological compatibility with standard cheesemaking (*34*, *55*).

Fresh cheese fortified with olive leaf powder further confirmed the enrichment potential of olive-derived ingredients, showing substantial increases in phenolics (up to 65 %) and antioxidant activity, while maintaining acceptable sensory properties at low contents (*17*). Conversely, the impact of thyme powder was more matrix-specific. In White Syrian cheese, higher thyme content increased total solids and salt uptake and led to higher acidity during storage. The combination of increased salt and thyme powder contents slowed ripening by reducing protein degradation and soluble nitrogen, demonstrating a distinctive modulatory effect on cheese maturation (*59*).

Research on GWHE has so far been limited to model systems or non-dairy foods, where strong antioxidant and antimicrobial effects have been documented, but its application in real cheese matrices has not been reported. This absence likely reflects both the intense pigmentation of the extract, as well as its astringency and high tannin content, which may challenge sensory acceptability of pale cheeses, as well as a general lack of technological trials in this specific product category.

Overall, these findings indicate that plant extracts, particularly OLE, can be strategically applied during cheesemaking to enhance antioxidant properties, microbial stability and textural attributes while maintaining or improving overall quality. Differences in extract type, content and incorporation stage determine the magnitude and nature of these effects, underscoring the importance of process optimisation for successful formulation of functional cheese products.

## REGULATORY ASPECTS AND SAFETY OF PLANT EXTRACT USE

The use of plant-derived extracts in foods is governed by strict regulatory frameworks designed to ensure consumer safety, product quality and consistency. These regulations differ between jurisdictions and depend largely on how the extract is classified, whether as a food ingredient, food additive, dietary supplement, novel food or medicinal preparation. Because botanical extracts often contain complex mixtures of bioactive compounds, their approval requires detailed characterisation, toxicological evaluation and evidence of safe use. These requirements are particularly relevant for functional dairy products, where plant extracts are incorporated not only for technological improvements but also for their antioxidant, antimicrobial and health-promoting potential.

The regulatory status of plant extracts differs considerably across jurisdictions and hinges on product classification. In the European Union, extracts intended for food are subject to the Novel Foods Regulation when they lack a history of significant consumption prior to 15 May 1997; such applications must include compositional, toxicological and exposure data (*60*). EFSA guidance for botanicals and botanical preparations used in supplements further emphasises identity verification, contaminant control and standardised marker content. Nutrition and health claims require separate EFSA evaluation under EU health-claims rules. In addition to novel food and safety requirements, the use of extraction solvents must comply with EU legislation on extraction solvents used in the production of foodstuffs and food ingredients. Directive 2009/32/EC (*61*) defines permitted extraction solvents and, where applicable, maximum residue limits in food products. This is particularly relevant for plant extracts obtained using organic solvents such as ethanol, methanol, acetone or other solvent systems, as residual solvent content must be controlled to ensure consumer safety and regulatory compliance. Therefore, solvent selection for plant extracts intended for dairy applications should consider not only extraction efficiency and selectivity, but also food-grade status, residue limits and suitability for incorporation into food matrices.

In the United States, botanical ingredients used in conventional foods fall under the food additive or GRAS frameworks (*62*). For dietary supplements, a New Dietary Ingredient (NDI) notification is required for extracts not marketed before 15 October 1994. FDA also provides dedicated guidance for botanical drugs when extracts are intended for medicinal use.

Regulatory expectations include robust characterisation (botanical identity, plant part used, extraction solvent), contaminant testing (pesticides, heavy metals, PAHs, mycotoxins), stability data and human exposure assessment. Toxicity and allergenicity remain key considerations: olive leaf extracts and oleuropein generally show high acute LD_50_ values (>1000–2000 mg/kg in animal studies), while thymol exhibits moderate acute toxicity (LD_50_≈1000 mg/kg) and may cause irritation. Juglone, a naphthoquinone characteristic of green walnut husk, is a known irritant capable of causing contact dermatitis, and at high concentrations botanical extracts may show cytotoxicity in model systems (*63*).

Herb–drug interactions and allergic reactions also require consideration. Certain botanical constituents may modify pharmacokinetics or pharmacodynamics, *e.g*. thyme components may influence coagulation and potentiate anticoagulants, while olive leaf compounds may affect blood pressure regulation (*64*). For these reasons, regulatory dossiers and product labels must address contraindications, allergenic potential and interaction risks.

In Croatia, national regulations align with EU legislation, particularly Regulation (EU) 2015/2283 on Novel Foods (*65*) and Regulation (EC) 1333/2008 on food additives (*66*). Plant extracts without a documented history of consumption in the EU prior to 15 May 1997 require full safety assessment, including characterisation, contaminant analysis and exposure evaluation. The Croatian Ministry of Health oversees notifications for extracts used in foods and supplements, requiring data on standardised markers, contaminants and stability. Particular attention is paid to heavy metals, pesticides, mycotoxins and PAHs. Olive leaf and thyme extracts are commonly used in supplements, while green walnut husk extract requires additional caution due to the presence of juglone. Product labelling must comply with consumer information requirements, including allergenicity and potential interactions (*67*, *68*).

Although regulatory frameworks permit the use of plant extracts in functional foods, strict requirements for identity, purity and safety testing may slow innovation and market entry, underscoring the need for clearer, harmonised guidelines for the safe application of botanicals in food systems.

In summary, while regulatory systems provide essential safeguards for consumer protection, their complexity and variability pose challenges for the development of plant-enriched functional dairy products. Ensuring compliance with compositional, toxicological and labelling requirements is particularly important for extracts containing potent bioactive compounds such as oleuropein, thymol or juglone. Future progress in this field will benefit from more harmonised regulatory approaches and clearer criteria for assessing botanical extracts intended for incorporation into food matrices, thereby facilitating safe and efficient innovation within the functional dairy sector.

## CONCLUSIONS

This review highlights the strong potential of olive leaf and thyme extracts as natural, polyphenol-rich ingredients for functional dairy applications, while green walnut husk extract shows emerging potential that requires further validation in real dairy matrices. Their incorporation into fermented and non-fermented dairy products may improve antioxidant capacity, microbial stability and technological performance, while sustainable extraction techniques support the valorisation of agro-industrial by-products. However, challenges remain, including sensory limitations at higher contents, variability in extract composition, limited data on green walnut husk application in real dairy systems, and the need for a deeper understanding of polyphenol–protein interactions and *in vivo* bioavailability. A limitation of this review is the heterogeneity of the available literature, including differences in extract composition, extraction methods, dose expression, dairy matrices and analytical approaches. These differences limit direct comparison among studies and make it difficult to draw firm conclusions regarding efficacy across product types.

Future research should focus on standardised extract characterisation, dose optimisation in specific dairy matrices, sensory threshold determination, evaluation of polyphenol stability and bioavailability during digestion, and well-designed *in vivo* or human intervention studies to confirm potential physiological effects. Particular attention should be given to green walnut husk extract, for which validation in real dairy matrices remains limited. Addressing these gaps, alongside navigating complex regulatory requirements for botanical ingredients, will be essential to enable wider industrial adoption of plant extract-enriched dairy products that align with clean-label and sustainability trends.
